# Experimentally heat‐induced transposition increases drought tolerance in *Arabidopsis thaliana*

**DOI:** 10.1111/nph.18322

**Published:** 2022-07-09

**Authors:** Michael Thieme, Arthur Brêchet, Yann Bourgeois, Bettina Keller, Etienne Bucher, Anne C. Roulin

**Affiliations:** ^1^ Department of Plant and Microbial Biology University of Zurich 8008 Zürich Switzerland; ^2^ Department of Environmental Sciences – Botany University of Basel 4056 Basel Switzerland; ^3^ School of Biological Sciences University of Portsmouth PO1 2DT Portsmouth UK; ^4^ Crop Genome Dynamics Group Agroscope 1260 Nyon Switzerland

**Keywords:** adaptation, *Arabidopsis thaliana*, drought tolerance, experimental evolution, loss‐of‐function mutation, transposable elements

## Abstract

- Eukaryotic genomes contain a vast diversity of transposable elements (TEs). Formerly often described as selfish and parasitic DNA sequences, TEs are now recognised as a source of genetic diversity and powerful drivers of evolution. However, because their mobility is tightly controlled by the host, studies experimentally assessing how fast TEs may mediate the emergence of adaptive traits are scarce.
- We exposed *Arabidopsis thaliana* high‐copy TE lines (hcLines) with up to *c.* eight‐fold increased copy numbers of the heat‐responsive *ONSEN* TE to drought as a straightforward and ecologically highly relevant selection pressure.
- We provide evidence for increased drought tolerance in five out of the 23 tested hcLines and further pinpoint one of the causative mutations to an exonic insertion of *ONSEN* in the *ribose‐5‐phosphate‐isomerase 2* gene. The resulting loss‐of‐function mutation caused a decreased rate of photosynthesis, plant size and water consumption.
- Overall, we show that the heat‐induced transposition of a low‐copy TE increases phenotypic diversity and leads to the emergence of drought‐tolerant individuals in *A. thaliana*. This is one of the rare empirical examples substantiating the adaptive potential of mobilised stress‐responsive TEs in eukaryotes. Our work demonstrates the potential of TE‐mediated loss‐of‐function mutations in stress adaptation.

## Introduction

Plants are constantly exposed to fluctuating environments. To successfully reproduce, they rely on mechanisms that allow them to react and adapt to suboptimal growth conditions. Genetic variation is a prerequisite for adaptation and the evolution of new traits. There is evidence that severe stresses can not only trigger the formation of small‐scale mutations (Belfield *et al*., 2021; Lu *et al*., 2021) but also increase genetic diversity through the stress‐induced activation of transposable elements (TEs) (McClintock, 1950; Lisch, 2013; Negi *et al*., 2016). In eukaryotic genomes, TEs are highly abundant (Wells & Feschotte, 2020) and the proportion of TE‐derived sequences in plants can reach up to > 80% in cases of some crops such as maize (Stitzer *et al*., 2021) or wheat (Wicker *et al*., 2018). In contrast with single nucleotide polymorphisms (SNPs), TEs can not only efficiently knock‐out genes (Van Houwelingen *et al*., 1998; Ram *et al*., 2019) but also bring their flanking regions under epigenetic control or render them stress responsive (Butelli *et al*., 2012; Grandbastien, 2015; Makarevitch *et al*., 2015; Roquis *et al*., 2021). By moving in response to the environment, TEs are therefore believed to be of particular importance for generating the phenotypic diversity needed for rapid adaptation to challenging environments (Torkamanzehi *et al*., 1992; Walser *et al*., 2006; Naito *et al*., 2009; González *et al*., 2010; Hof *et al*., 2016; Rey *et al*., 2016; Li *et al*., 2018; Esnault *et al*., 2019; Baduel *et al*., 2021).

In plants, the majority of TEs are class I elements belonging to the subclass of long terminal repeat (LTR)‐retrotransposons that move through the reverse transcription of an RNA‐intermediate in a copy‐and‐paste mechanism (Wicker *et al*., 2007; Schulman, 2013). To ensure a limited mutation rate and to safeguard genome stability, TE mobility is usually restricted by epigenetic silencing mechanisms, which in plants involves the RNA‐directed DNA methylation (RdDM) pathway (Matzke & Mosher, 2014). As a consequence, only few TE families have been observed transposing *in planta* (McClintock, 1953; Grandbastien *et al*., 2005; Picault *et al*., 2009; Lanciano *et al*., 2017; Masuta *et al*., 2018; Benoit *et al*., 2019) and this even in plants free of DNA methylation (He *et al*., 2022). The understanding to what extent TEs may play a role in adaptation in this kingdom therefore largely relies on population genomics data and correlative studies (Quadrana *et al*., 2016; Li *et al*., 2018; Stritt *et al*., 2018; Baduel *et al*., 2021) rather than on experimental validation.

In this context, we study here the functional impact of the 5 kb *Arabidopsis thaliana* retrotransposon *ONSEN* (*AtCOPIA78*), one of the best characterised TE families in plants. Equipped with heat‐responsive elements in its LTRs, *ONSEN* can sense the heat stress‐response of its host and utilise it to initiate its own copy‐and‐paste lifecycle (Tittel‐Elmer *et al*., 2010; Ito *et al*., 2011; Cavrak *et al*., 2014). While transgenerational transposition events of *ONSEN* in wild‐type (wt) plants are too rare to be observed in real‐time (Hayashi *et al*., 2020), transcripts and reverse transcribed extrachromosomal DNA copies of *ONSEN* in the Col‐0 wild‐type (wt) can already be detected following a heat shock at 37°C for 12 h (Ito *et al*., 2011; Cavrak *et al*., 2014). Moreover, genomic copy numbers of *ONSEN* are known to vary between natural accessions of *A. thaliana* (Masuda *et al*., 2016; Quadrana *et al*., 2016) suggesting an ongoing heat‐dependent mobility of *ONSEN* in wild populations.

To overcome the limitation of the low transposition frequency of active TEs in natural accessions, we previously developed a method to amplify *ONSEN* in wt plants. By transiently reducing TE silencing by the inhibition of DNA‐methyltransferases (Baubec *et al*., 2009) and RNA polymerase II in combination with a heat shock for 24 h at 37°C, we were able to increase *ONSEN* activity, which resulted in the integration and inheritance of novel genomic *ONSEN* copies in *Arabidopsis* Col‐0 wt plants (please refer to Materials and Methods section and Thieme *et al*., 2017). The copy numbers of the selected *ONSEN* high‐copy TE lines (hcLines) were stable over three generations of selfing suggesting genetic stability (Thieme *et al*., 2017). Notably, the distribution of novel *ONSEN* copies in these hcLines is not random but reflects a distinct insertion bias towards exons and H2A.Z enriched regions (Roquis *et al*., 2021) confirming previous findings in TE accumulation lines (Quadrana *et al*., 2019).

The transposition of *ONSEN* is known to result in transcriptomic changes (Ito *et al*., 2011; Roquis *et al*., 2021) and new phenotypes such as altered seed colour (Thieme *et al*., 2017) or abscisic acid insensitivity (Ito *et al*., 2016). However, while the heat‐dependent mobility of *ONSEN* could therefore create the raw material for evolution and was proposed to confer a unique adaptive potential to global warming in *A. thaliana* (Quadrana *et al*., 2016), its immediate adaptive potential and fitness effects have not been tested experimentally. Due to climate change and temperature increase, drought is predicted to constitute one of the most severe environmental constraints to which plants will have to adapt in the near future (Exposito‐Alonso *et al*., 2019; Brás *et al*., 2021). We used here our collection of hcLines to experimentally test whether and how the heat‐induced transposition of *ONSEN* may help individuals to survive in warmer and therefore water‐limited environments.

## Materials and Methods

### Plant material

*ONSEN* hcLines were generated by treating *A. thaliana* Col‐0 plants with a combination of a heat shock and drugs that inhibit TE silencing, as described previously (Thieme *et al*., 2017). Briefly, Col‐0 seeds were germinated and grown under long day conditions (16 h light) at 24°C (day) 22°C (night) on half‐strength Murashige and Skoog (½MS) medium with 1% sucrose and 0.5% Phytagel, pH 5.8. To reduce TE silencing and increase the rate of *ONSEN* transposition, seedlings were grown analogously on ½MS medium supplied with a combination sterile filtered zebularine (Z, 40 μM) and α‐amanitin (A, 5 mg ml^−1^). After 7 d of growth on control ½MS or medium supplied with A and Z, seedlings were ether exposed to control stress (CS; 24 h at 6°C followed by 24 h at normal conditions) or heat stress (HS; 24 h at 6°C followed by 24 h at 37°C), then transferred to soil and selfed to obtain the S1 generation. Individual S1 plants originating from plants that were either only exposed to CS or HS or additionally treated with A and Z (AZ) were separated and repeatedly self‐fertilised until we obtained the S4 generation. In this study we used 23 *ONSEN* hc‐lines originating from 13 plants that were treated with HS + AZ, five independent control lines that were either only exposed to CS (two lines), HS (two lines) or CS + AZ (one line) and the Col‐0 wild‐type that was propagated on soil. The *rpi2‐1* mutant (SALK_022117) (Xiong *et al*., 2009) was obtained from the Nottingham Arabidopsis Stock Centre (Alonso *et al*., 2003).

### qPCR for *ONSEN* copy numbers

To determine the average *ONSEN* copy numbers of the hcLines and controls used in this study, we extracted DNA of the aboveground parts of at least 24 pooled individuals per line of the S4 generation grown for 8 d under sterile conditions on ½MS medium (1% sucrose, 0.5% Phytagel, pH 5.8) under long day conditions (16 h light) at 24°C (day) 22°C (night) using the DNeasy Plant Kit (Qiagen). *ONSEN* copy numbers were determined by qPCR using 12 ng total DNA using the KAPA SYBR FAST master mix universal on a C1000 Touch (Bio‐Rad) machine. *ACTIN2* (*At3g18780*) was used to normalise DNA levels and DNA of Col‐0 served as a control. Three technical replicates were used and data were analysed using the Bio‐Rad CFX Manager 3.1 software. Sequences of oligos are listed in Supporting Information Table S1.

### Identification, expression analysis and visualisation of *ONSEN* and T‐DNA insertions

Novel *ONSEN* insertions of hcLine31 were identified and characterised recently (Roquis *et al*., 2021) by whole‐genome sequencing. Briefly, reads were trimmed using trimmomatic (Bolger *et al*., 2014) with the parameters ILLUMINACLIP: TruSeq3:2:30:10 LEADING:20 TRAILING:20 SLIDINGWINDOW:4:20 and MINLEN:36. *ONSEN* insertions were detected and analysed previously using Transposable Insertion Finder v.1.6 (Nakagome *et al*., 2014) with the TAIR10 version of the *A. thaliana* Col‐0 reference genome (Berardini *et al*., 2015) as described in detail (Roquis *et al*., 2021). Processed genomic reads were mapped to the TAIR10 version of the *A. thaliana* genome using bwa mem (v.0.7.17‐r1188) (Li & Durbin, 2009) with the ‐M parameter set. To validate the presence and zygosity of the *ONSEN* and T‐DNA insertions in *RPI2* in the segregating F2 populations, we designed primers (mto_007 and mto_067) spanning the predicted insertion sites of *ONSEN* and the T‐DNA (based on SIGnAL) and combined them with primers specific to *ONSEN* (mto_196) or the T‐DNA (LBb1.3 mto_063) (Fig. 3a; Table S1). For the PCRs we used a standard *Taq* DNA polymerase (Sigma Aldrich) and limited the elongation time to 90 s so that an homozygous insertion of the 5‐kb *ONSEN* TE or the T‐DNA would prevent the formation of a PCR product. To genotype the F2 populations, we used DNA from the homozygous parental plants (wt, hcLine31 and *rpi2‐1* (SALK_022117)).

RNA‐seq data of one representative biological replicate of the Col‐0 wt and hcLine31 exposed to CS and whole‐genome sequencing data of hcLine31 were obtained from and analysed according to a previous report (Roquis *et al*., 2021). Briefly, reads were trimmed using trimmomatic (Bolger *et al*., 2014) as described above. Processed RNA‐seq reads were aligned to the TAIR10 version of the *A. thaliana* genome using Star (v.2.7.9a) (Dobin *et al*., 2013) using the same parameters such as Roquis *et al*. (2021) but with ‐alignIntronMax set to 10 000. The insertion site of *ONSEN* was visualised using the generated bam files and the packages gviz (v.1.28.3) (Hahne & Ivanek, 2016), rtracklayer (v.1.44.4) (Lawrence *et al*., 2009) and the annotation package txdb.athaliana.biomart.plantsmart28 (v.3.2.2) (Carlson & Maintainer, 2015) in R (v.3.6.3) (R Core Team, 2020) in Rstudio (v.1.1.456) (RStudio Team, 2016).

### Drought assay

To obtain comparable and robust results, we ran one comprehensive drought experiment in which we tested the S4 generation of hcLines, the control lines and the segregating F2 generations of crosses between hcLine31 and Col‐0, and hcLine31 and *rpi2‐1* (SALK_022117) in parallel. We included five replicates for each high‐copy and control line and for the parents of the cross between *rpi2‐1* and hcLine31, and tested 22 F_2_ individuals of the cross of hcLine31 with the wt and 16 F_2_ individuals of the cross of hcLine31 and *rpi2‐1* (SALK_022117). Seeds were sown in pots filled with Einheitserde that was incubated with a solution (75 mg l^−1^) of the insecticide Kohinor (Leu + Gygax AG) and kept at 4°C for 3 d. After stratification, pots were moved into a Hiros climate chamber (Clitec GmbH, Küssnacht am Rigi, Switzerland) set to short day conditions with 10 h light (LED Valoya Ns12 C75/65, *c*. 120 μmol m^−2^ s^−1^) at 22°C : 19°C, day : night, with 60% humidity. After 10 d of growth, seedlings were picked into pots filled with equal amounts of Kohinor‐treated soil and grown under well watered conditions for 36 d. The position of pots was frequently shifted to ensure similar growth conditions. Before watering was suspended, pots were again saturated with water and weighted to obtain the maximal water content. At 1 d later (day 1 of the experiment), top‐view pictures were taken with a Canon EOS 70D camera on a tripod at the following settings: 5.6 s shutter opening, 1/60 shutter speed, ISO 200. This procedure was repeated three times at an interval of 7 d until day 22 of the experiment. Due to technical issues, nine out of 193 images (affecting one to two biological replicates of seven different lines and one biological replicate of the F2 of the cross of hcLine31 and *rpi2‐*1) from day 15 are missing in the analysis. On day 27, pots were again weighted, top‐view pictures were taken, and drought stress was stopped by filling trays with water and allowing the pots to absorb water over night. After 2 d of regeneration under well watered conditions, final pictures of the plants were taken (day 29). To account for different zoom levels during the course of the experiment, we took photographs of a white label that later served as a calibrator to normalise predicted vital and necrotic leaf areas. At 1 d after the last photographs were taken, one leaf of each plant from the segregating F2 populations was sampled for DNA extractions and genotyping. Pots were removed from the climate chamber and dried for 8 wk at room temperature to obtain the dry weight to calculated the water content of each pot. Pictures and weight were determined on 2 d successively (except for day 27); therefore, we extrapolated the weight measurements to determine the water content on the exact day pictures were taken.

### Machine‐learning‐based prediction of necrotic and vital leaf areas

We used the pixel classification tool of Ilastik (v.1.3.3post3) (Berg *et al*., 2019) with all 13 features for colour/intensity, edge, texture of sigma 0.3, 0.7 and 10.00 selected. We defined three different pixel classes: ‘background’, ‘necrotic’ and ‘vital’. We performed an iterative manual training to gradually improve the accuracy of the prediction and finally used 24 images of plants at different stages of the experiment to train the model. For the disc assay, we combined one to three leaf discs that were punched from vital and necrotic leaves onto soil of a single pot that did not contain a plant. We took three photographs of each combination. To cover a broad spectrum of possibilities, leaf discs were shuffled and/or moved on the pot between pictures. Therefore some discs were photographed multiple times but in different combinations and/or positions. Similar to the model training with living and necrotic tissues, we used five images to train ilastik to detect background and the white labels that served as a scale to normalise pixel counts between different days of the experiment. We then processed and exported all image files in ilastik with the following settings: source, ‘simple segmentation’; convert to datatype, ‘floating 32 bit’; format, ‘tif’, and used the ‘getHistogram’ function of ImageJ (v.1.53g, Java 1.8) (Schneider *et al*., 2012) to extract the pixel counts of the areas predicted by ilastik. Pixel counts of vital and necrotic leaves were then normalised using the predicted areas of the size references for each time point of the drought experiment using R.

### SPAD value and C : N ratio

We grew S4 generation plants under the same conditions and watering regime as for the drought experiment and used a chlorophyll meter SPAD‐502 chlorophyll meter (Ling *et al*., 2011) to determine the Soil Plant Analysis Development (SPAD) values of three or six leaves of six wt and hcLine31 plants that were grown as triplicates in two independent experiments. SPAD values were measured before the occurrence of necrotic leaves 15 d after watering was suspended.

To determine the carbon/nitrogen ratios of the wt and hcLine31 we grew S3 generation plants under well watered conditions on Einheitserde under short day conditions in a Sanyo MLR‐350 growth chamber with an 8 h : 16 h, 20°C : 18°C, day : night cycle for 14 wk. We sampled one leaf from each of four plants of the wt and hcLine31, inactivated them for 30 s in a microwave and dried them for 8 d at 60°C. Plant material was then ground for 3 min at 30 Hz with an oscillating mill (MM400; Retsch GmbH, Haan, Germany). Then, 2 mg of plant material were put in tin capsule and C : N ratios were analysed with a thermal conductivity detector by the Basel Stable Isotope Laboratory.

### *RPI2* analysis in natural accessions

We used the vcf file produced by The 1001 Genomes Consortium (Alonso‐Blanco *et al*., 2016) to extract SNPs for 1135 sequenced accessions of *A. thaliana*. To limit the effect of the phylogenetic relationship in further analyses, we used the function ‘–relatedness2’ from vcftools (Danecek *et al*., 2011) to keep only ecotypes with a kinship coefficient *k* < 0.5. For the remaining ecotypes, bioclimatic variables (https://www.worldclim.org/data/worldclim21.html) and Global Aridity Index (https://cgiarcsi.community/data/global‐aridity‐and‐pet‐database/) were extracted using the R packages raster (v.3.5‐2 (Hijmans & van Etten, 2012)) and rgdal (v.1.5‐27 (Keitt *et al*., 2010)). We fitted a linear mixed model with the R package lme4 (v.1.1‐27 (Bates *et al*., 2015)) to test the association between aridity levels averaged over May to August and SNPs in *RPI2* harbouring a minor allele frequency (maf) > 0.3. We added the admixture groups defined by The 1001 Genomes Consortium (Alonso‐Blanco *et al*., 2016) as a random effect to account for population structure. We used Qgis (v.3.16; https://www.qgis.org/en/site/) to display *RPI2* alleles in Eurasia. For the rest of the manuscript, all statistical analyses were performed in R and parametric or nonparametric tests were used according to the sample size and data distribution.

### Survival probability of *ONSEN* in *RPI2*

TIPs in *RPI2* were screened in the data published by Baduel *et al*. (2021). We used forward‐in‐time models to examine the short‐term fate of a TE inserting in *RIP2*, simulating a population of 100 diploid individuals undergoing drought events. We used the SliM3 simulator (Haller & Messer, 2019) to model a population with a selfing rate of 99% (Richard *et al*., 1989), and ran the simulations for 50 generations. Assuming a generation time of *c*. 2 months and 6 months of suitable growth conditions per year, this simulation should cover *c*. 16 yr of a natural population. The TE insertion only had a fitness effect at the homozygous state (dominance coefficient *h* = 0) and was initially sampled during the first generation from a single genome (over 200). The selective advantage (*s*) of individuals carrying the TE insertion at the homozygous state during the drought events was set at +50%, +100%, +200% or +500% relative to the wild‐type. During normal conditions, this coefficient was set at −10%, −20%, −50% or −90% relative to the wild‐type. We applied a model of hard selection, with a population size being a function of the average fitness, with a maximal population size of 100 (carrying capacity). This was included as a way to consider the varying effects of drift in small populations, which may affect the odds for a deleterious allele to rise in frequency. We simulated one to four drought events at regular intervals. Each of these events could last two, three or four generations. We ran 1000 simulations for each combination of parameters. Scripts are freely available at https://github.com/YannBourgeois/Slim‐simu‐TE.

## Results

### *ONSEN* hcLines differ in growth

**Fig. 1:**
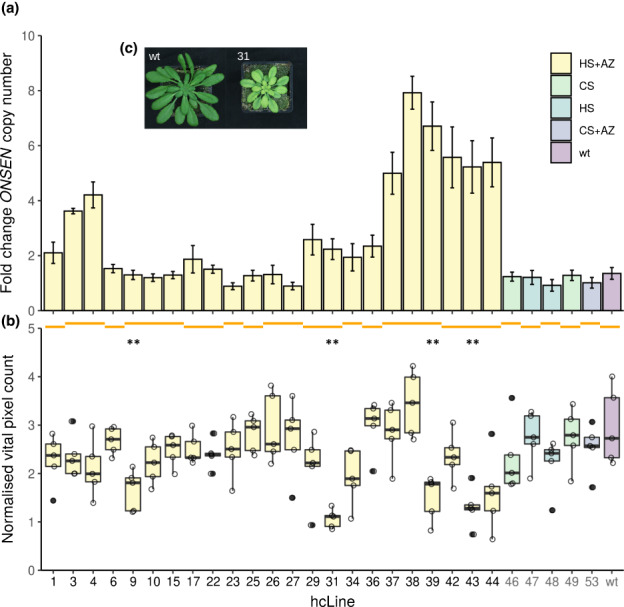
*ONSEN* copy numbers and size of *Arabidopsis thaliana* hcLines. Box colours indicate the history of the lines: control stress (CS), heat stress (HS), chemical de‐methylation (AZ) and wild‐type propagated on soil (wt). Orange lines spanning multiple hcLines indicate their origin from a common parent. Control lines and the wt are indicated by grey tick labels. (a) Fold change of *ONSEN* copy numbers measured by qPCR compared with the wt (*n* = 3 technical replicates ±SD). (b) Vital pixel count of hcLines and controls before the occurrence of necrotic leaves (day 8). Significant differences to the wt are indicated. Horizontal line defines median, hinges represent 25^th^ and 75^th^ percentiles, whiskers extend to 1.5× IQR and outliers are shown as filled dots. *n* = 5 biological replicates. Wilcoxon test: **, *P* < 0.01. (c) Representative pictures of the wt and hcLine31 showing significant differences in size (please refer to (b)) on day 8 of the experiment.

We grew the S4 generation of 23 of the aforementioned hcLines originating from 13 independent heat stressed and transiently de‐methylated Col‐0 plants (please refer to Materials and Methods section and Thieme *et al*., 2017) and validated by quantitative PCR (qPCR) an up to *c*. 8 fold copy number increase (therefore up to *c*. 64 stably inserted *ONSEN* copies; Fig. 1a) compared with the wt. However, in some hcLines (e.g. hcLine9), no additional *ONSEN* copies were detectable by qPCR even though some were previously validated by sequencing (Roquis *et al*., 2021) indicating a higher sensitivity of the TE‐detection pipeline compared with the qPCR.

As the hcLines were originally exposed to a combination of heat and the drugs zebularine (Z) and alpha‐amanitin (A) (HS + AZ) (please refer to the Materials and Methods section and Thieme *et al*., 2017), we harnessed a set of control lines to account for a potential *ONSEN*‐independent phenotypic variation caused by epi/genetic changes induced by the heat stress or the chemical demethylation. Namely, we included a Col‐0 wt plant that was propagated on soil and five independent controls, that is lines that originated from plants that were also grown *in vitro* but only exposed to CS (two lines), HS (two lines) or CS plus chemical demethylation (CS + AZ; one line) (Thieme *et al*., 2017). In accordance with previous observations (Thieme *et al*., 2017; Roquis *et al*., 2021), and the strict heat dependence of *ONSEN* (Ito *et al*., 2011; Cavrak *et al*., 2014) we did not detect an increase in genomic *ONSEN* copy numbers in these control lines when compared with the wt (Fig. 1a).

**Fig. 2:**
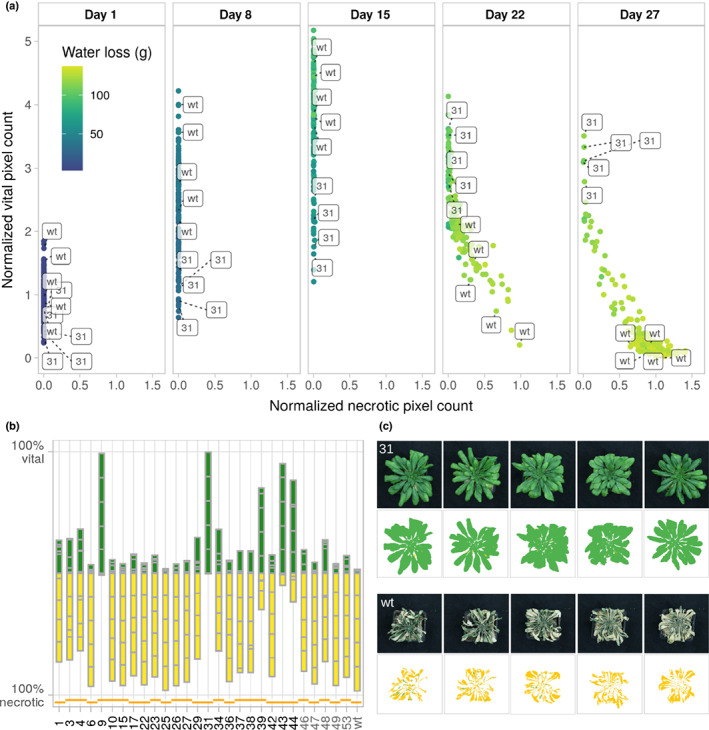
Drought tolerance of *Arabidopsis thaliana ONSEN* hcLines. (a) Pixel counts of living and necrotic tissues of the six control and 23 *ONSEN* high‐copy lines a during the drought stress. Each dot represents one individual plant and the colour code indicates the cumulative water loss over time. *n* = 3–5 biological replicates per line are shown. (b) Percentage of vital (green) and necrotic (yellow) tissues of all five replicates per line 2 d after recovery (day 29). The contribution of each replicate to the total amount of vital and necrotic pixels is indicated with grey bars. Orange lines spanning multiple hcLines indicate their origin from a common parent. Control lines and the wt are indicated by grey tick labels. (c) Original (upper) and processed (ilastik, lower) images of the five replicates of hcLine31 (31) (upper) and wt (lower) on day 29. Predicted vital leaf segments are depicted in green, necrotic segments in yellow.

We suspended watering after 36 d of growth and recorded plant development and water loss of the pots every week by taking top‐view pictures and by weighting the pots. To quantify the growth and the degree of drought‐induced leaf senescence, we trained the image‐based interactive learning and segmentation toolkit (ilastik) (Berg *et al*., 2019) to specifically detect living (from this point forwards vital) and necrotic leaf segments. We first tested the accuracy of the prediction by placing one to three punched leaf discs of necrotic or vital segments onto a pot that did not contain a plant. After processing the images with ilastik, we obtained a linear increase of vital and necrotic pixel counts according to the number of segments placed onto the pots (Fig. S1), confirming the reliability of the method. For the rest of the study, we therefore used vital pixel counts as a proxy for plant size. To assess size variations between the hcLines, we analysed the pixel counts of predicted vital areas before the appearance of necrotic leaves 8 d after watering was suspended (Figs 1b, 2a). Although heat stress and the AZ drug treatment have been shown to induce epi/genetic mutations (Liu *et al*., 2015; Belfield *et al*., 2021; Roquis *et al*., 2021), we did not observe differences in growth among our five control lines. However, in accordance with the fact that transposition is predominantly associated with fitness loss of the host (Wilke & Adams, 1992; Boissinot *et al*., 2006; Chuong *et al*., 2017; Roquis *et al*., 2021), we found a significantly reduced number of vital pixels compared with the wt for four hcLines (Wilcoxon test *P* < 0.01; Fig. 1b,c).

Notably, in some cases we also observed a strong size variation between hcLines originating from the same parent (e.g. hcLine37 and hcLine38 vs hcLine39) indicating an expected (Matsunaga *et al*., 2015) and previously reported (Roquis *et al*., 2021) genetic segregation of the lines (Fig. 1b). We did not find any significant global correlation between *ONSEN* copy numbers and plant size (*P* > 0.05, Fig. S2). Taken together, these observations suggest that single TE insertions rather than the overall *ONSEN* load were responsible for the observed phenotypic variation.

### An *ONSEN* ‐mediated loss‐of‐function mutation leads to an increased drought tolerance of hcLine31

The machine learning approach allowed us to further track the dynamics of growth and drought‐induced necrosis of the individual plants. None of the plants had necrotic leaves until 15 d after watering was suspended, (Fig. 2a). After 27 d, we resumed watering and recorded leaf areas after 2 d of recovery (day 29). In contrast with the high drought‐induced mortality of the five control lines and the wt, five out of the 23 hcLines (hcLine9, 31, 39, 43 and 44) originating from four independent parental lines were more stress tolerant (mean vital leaf area > 50%) (Fig. 2b). Although the water limitation was lethal for the wt (0.03% mean vital area), hcLine31 did not show any signs of necrosis (97.9% mean vital area, Fig. 2a–c). Because hcLine31 displayed the most drought‐tolerant phenotype (Fig. 2b,c), we selected it to characterise the functional link between the heat‐induced insertion of novel *ONSEN* copies and the observed increase in drought tolerance. Note that because wt plants and many hcLines died from drought stress before the end of the experiment, no other fitness‐related traits (e.g. seed production) were assessed.

We then harnessed whole‐genome re‐sequencing data that had been previously used to locate all transposon‐insertion polymorphisms (TIPs) of hcLine31 (Roquis *et al*., 2021). In accordance with its insertion bias towards actively transcribed regions in the *A. thaliana* genome (Quadrana *et al*., 2019; Roquis *et al*., 2021), six out of the 10 *ONSEN* TIPs detected in hcLine31 were located in exons (Table 1). The consistent vitality of all five replicates of hcLine31 indicated that a homozygous mutation was underlying the observed drought tolerance of hcLine31. We also speculated that the causal insertion was located in the exon of a gene. Therefore, we considered only three (in *At1G58602*, *At2G01290* and *At5G03435*) out of the 10 novel *ONSEN* copies as candidate insertions. Based on our phenotypic observation, we selected the insertion in the *ribose‐5‐phosphate‐isomerase 2* (*RPI2, At2G01290*) as our top candidate, as *RPI2* is involved in chloroplast photosynthetic capacity (Xiong *et al*., 2009). The link between drought stress on plant photosynthesis is indeed well documented (e.g. Reddy *et al*., 2004) and we therefore tested experimentally whether the mutation of *RPI2* was underlying the high survival rate of hcLine31.

**Table 1:** Novel *ONSEN* insertions in *Arabidopsis thaliana* hcLine31.

chr	Coordinates	Context	ID	Description	Zygosity
1	21761950–55	Exon	*AT1G58602*	LRR and NB‐ARC domains‐containing disease resistance protein	(−/−)
2	149387–91	Exon	*AT2G01290*	Cytosolic ribose‐5‐phosphate isomerase	(−/−)
3	19300993–97	Exon	*AT3G52020*	Serine carboxypeptidase‐like 39	(+/−)
3	22631755–59	Exon	*AT3G61150*	Homeodomain GLABROUS 1; HD‐ZIP IV family	(+/−)
5	853776–80	Exon	*AT5G03435*	Ca^2+^‐dependent plant phosphoribosyltransferase family protein	(−/−)
5	10632816–20	TE	*AT5G28626*	*AT5TE38720*; SADHU; Sadhu noncoding retroTE family	(−/−)
5	18850327–31	Promoter	*AT5G46490*	Disease resistance protein (TIR‐NBS‐LRR class) family	(−/−)
5	21050239–43	Intron	*AT5G51800*	Protein kinase superfamily protein	(−/−)
5	21602030–34	Exon	*AT5G53240*	Hypothetical protein (DUF295)	(+/−)
5	22846432–36	Intron	*AT5G56400*	FBD, F‐box, Skp2‐like and Leucine Rich Repeat domains‐containing protein	(+/−)

Location, description (Araport11) and zygosity (−/− homozygous *ONSEN*, +/− heterozygous) of predicted *ONSEN* insertion sites extracted from Roquis *et al*. (2021).

**Fig. 3:**
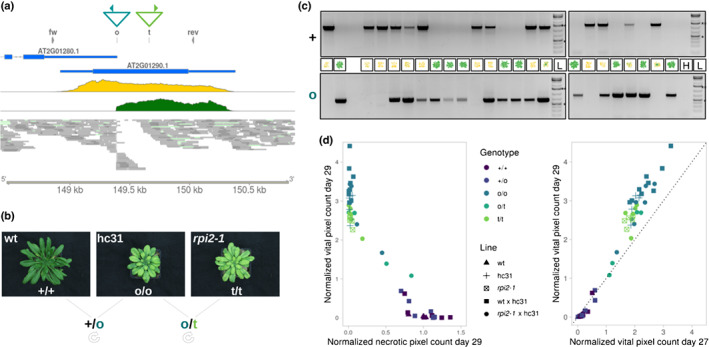
An *ONSEN* insertion in *RPI2* leads to an increased drought tolerance of *Arabidopsis thaliana* hcLine31 (hc31). (a) Triangles indicate the insertion site of *ONSEN* (turquoise) and the location of the T‐DNA in the *rpi2‐1* mutant (green) on chromosome 2. Primer locations used for the genotyping in (c) and (d) are depicted as filled triangles. Annotation track (blue), RNA‐seq coverage of the wt (yellow) and hcLine31 (green) and aligned genomic reads from hcLine31 are shown. (b) Images and crossing scheme of the wt, hcLine31 and *rpi2‐1*. Genotypes are depicted with + (wt), o (*ONSEN*), and t (T‐DNA). (c) Genotypes and phenotypes (day 29) of the segregating F2 population of hcLine31 × wt. A wt and a hcLine31 (first two lanes) are shown as references. Primers specific for the wt (upper gel, +) and the *ONSEN* insertion (lower gel, o) (please refer to (a)) were used. H, water control; L, GeneRuler 1 kb plus ladder. 0.5 kb (*) and 1.5 kb (**) bands are marked. (d) Pixel counts of living tissue after 2 d of recovery (day 29) in relation to necrotic tissues on the same day (left panel) and compared with vital pixel counts before recovery (day 27) (right panel). Shapes indicate the plant line (parental or segregating F2 individuals of the crosses from panel (b) and colours indicate the genotype of *RPI2*.

First, we verified the impact of the *ONSEN* insertion on the expression of *RPI2* by analysing previously published RNA‐seq data of hcLine31 (Roquis *et al*., 2021) and found a premature transcriptional stop coinciding with the detected exonic *ONSEN* insertion in *RPI2* (Fig. 3a). Knowing about the loss‐of‐function mutation of *RPI2* in hcLine31, we then crossed hcLine31 to a Col‐0 wt plant (Fig. 3b) and assessed the drought tolerance and genotypes of *RPI2* of individual plants in the segregating F2 generation obtained from self‐fertilisation. In accordance with our hypothesis that the loss‐of‐function of *RPI2* would lead to an increased drought tolerance of hcLine31, we found that solely F2 individuals carrying a homozygous *ONSEN* insertion survived the drought stress (Fig. 3c).

To conclusively verify these findings by combining the *ONSEN* insertion with another independent recessive allele of *rpi2*, we harnessed the mutant *rpi2‐1* (SALK_022117) that carries a homozygous T‐DNA insertion in the exon of *RPI2* that also leads to a knock‐out of the gene (Xiong *et al*., 2009). Accordingly, a cross between hcLine31 and the *rpi2‐1* mutant should only lead to drought‐tolerant offspring. Indeed, all F2 individuals of *rpi2‐1 x* hcLine31 that were either homozygous for the *ONSEN* or the T‐DNA insertion or that carried both the *ONSEN* and the T‐DNA‐insertions in *RPI2* survived the drought stress and showed continued growth 2 d after recovery (Fig. 3d). These results unequivocally demonstrated that the *ONSEN* insertion in *RPI2,* that results in a recessive loss‐of‐function mutation, was responsible for the increased drought tolerance of hcLine31.

### Reduced size and water use explains desiccation tolerance of hcLine31

**Fig. 4:**
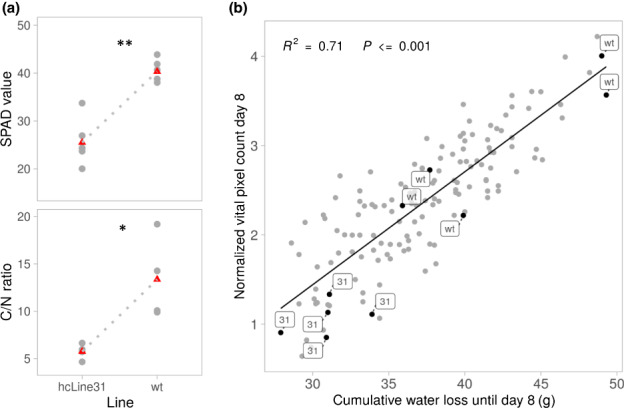
Physiological differences between *Arabidopsis thaliana* hcLine31 and wt plants. (a) SPAD meter values (upper) and C : N ratio (lower) of the two lines. *n* = 4–6 biological replicates. Means are marked with red triangles, Wilcoxon test: *, *P* < 0.05; **P < 0.01. (b) Relationship between pixel counts of living tissues and cumulative water loss until day 8 before the occurrence of necrotic leaves. Linear regression model, adjusted *R*‐squared: 0.71. *P* < 0.001.

As mentioned above a previous study indicated that the loss of *RPI2* leads to chloroplast dysfunction and reduced chlorophyll content (Xiong *et al*., 2009). To test whether the photosynthesis was indeed affected in hcLine31, we assessed carbon/nitrogen (C : N) ratios and SPAD values, which are directly linked to photosynthetic activity (Ling *et al*., 2011; Otori *et al*., 2017), following growth under well watered conditions and before the emergence of necrotic leaves. SPAD and C : N ratios were significantly reduced (Wilcoxon test, *P* < 0.05) in hcLine31 compared with the wt (Fig. 4a; Table S2). In accordance with (Xiong *et al*., 2009), this indicated a reduced photosynthetic capacity in hcLine31 before the onset of the drought stress. This raised a question about the mechanistic link between the *ONSEN* insertion in *RPi2* and the high survival rate under water limitation. As noted earlier, compared with the wt, we noticed a reduced size of hcLine31 under well watered conditions (Fig. 1b,c) and that drought‐induced symptoms in hcLine31 occurred significantly later (Fig. 2a). We therefore hypothesised that the hampered photosynthetic capacity resulting in a slower growth and therefore reduced water consumption of hcLine31 underlies the high survival rate of this line. Therefore we looked for a general link between plant size and drought tolerance. We first fitted a linear model in which water loss (before the occurrence of necrotic leaves at day 8 of the experiment) was entered as the response variable and the plant lines, vital pixel count and their interaction as explanatory variables. Although the overall model was significant and explained a large part of the variance (*R* ^2^ = 0.72, *P* < 2.2e−16), we did not detect a significant contribution of the plant line nor of the interaction between the line and vital pixel count, indicating that all hcLines, control lines and the wt had a similar efficiency regarding water use. However, we found that vital pixel count had a significant effect on water loss (*P* = 5.19e−11). This was further confirmed by a reduced model in which the line and interaction effects were dropped (Fig. 4b; *R* ^2^ 0.71, *P* < 0.001). Therefore, these analyses indeed suggested that the *ONSEN* insertion in *RPI2* resulted in reduced photosynthetic capacity leading to slower growth and a reduced water consumption, allowing hcLine31 to escape severe drought stress.

Based on these observations we also tested whether *RPI2* could play a more general quantitative role in the adaptation to arid climate in *A. thaliana* natural populations. We therefore further tested the link between *RPI2* and tolerance to aridity by extracting the Global Aridity Index in summer (GAI_summer_; Global Aridity Index averaged over April to August) for each locality of 596 genetically distinct *A. thaliana* ecotypes (kinship coefficient < 0.5 and no missing data for subsequent analyses) from the 1001 genome project (Alonso‐Blanco *et al*., 2016; Table S3).

**Fig. 5:**
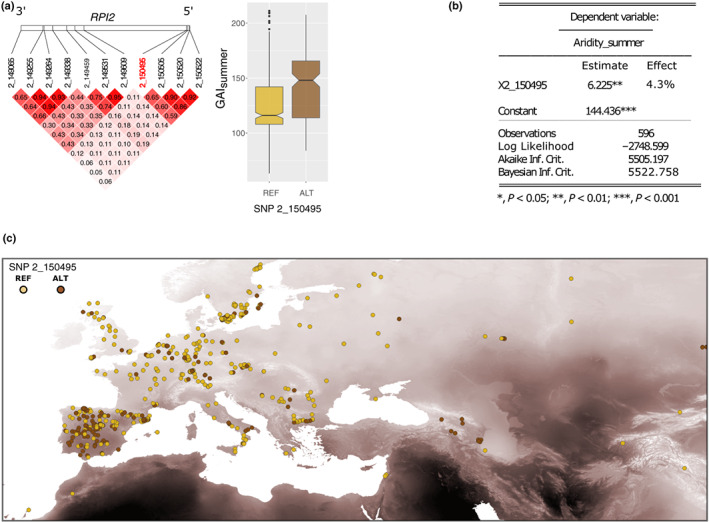
The association between aridity and *RPI2* variations in natural populations of *Arabidopsis thaliana*. (a) Linkage disequilibrium among the 11 SNPs located in *RPI2* (left panel) and the association between SNP 2_150495 and Global Aridity Index in summer (GAIsummer; right panel). (b) Linear mixed model results based on 596 accessions. (c) Occurrence of the reference and alternative alleles of SNP 2_150495 in Eurasia. The intensity of the background map displays aridity levels in July from low (light) to high (dark).

We identified 11 SNPs with a maf > 0.3 in *RPI2*. Although some of these SNPs were found to be in strong linkage disequilibrium (Fig. 5a), one of them located in the regulatory region of *RPI2* (chr2 at nt150495) showed a stronger association with GAI_summer_ (Fig. 5a). Our linear mixed model analysis revealed that this SNP was significantly associated with higher GAI_summer_ even when accounting for population structure (4.3% of the variance explained; Fig. 5b). Accordingly, we detected a high frequency of this allele in areas with high aridity levels in July (Fig. 5c). In summary, these findings suggest that our candidate *ONSEN* insertion in hcLine31 boosts the native function of *RPI2* with respect to adaptation to drought.

### Survival probability of *ONSEN* in *RPI2*

This type of insertion for *ONSEN* or other heat‐induced TEs could provide a selective advantage in the face of global warming (Ito *et al*., 2016; Quadrana *et al*., 2019; Baduel *et al*., 2021). However, because the knock‐out of *RPI2* leads to a reduced photosynthetic activity, one may expect large‐scale mutations providing such a strong effect on growth under well watered conditions to be purged by natural selection. In fact, we did not find any natural TE insertions in *RPI2* (Baduel *et al*., 2021). To further determine if and under which conditions large‐scale mutations with strong phenotypic effects could reach a high frequency in *A. thaliana* populations, we simulated different scenarios of temporary drought events (Fig. S3). Four parameters were applied to control the simulations: the number of drought events (one to four), the length of each drought event (two to four generations) and the selective advantage of the insertion during drought (s+) and normal conditions (s−). We used a model of ‘hard’ selection, in which the population size varied as a function of the average fitness. When drought events were rare and short, no TIP was found in the population after 50 generations. However, the frequency of the TIP increased with higher values of s+ and lower values of s−, but required frequent and long episodes of drought events. This confirmed that a mutation with high deleterious effects under optimal growth conditions is hardly maintained over time (Fig. S3). However, it also shows that this mutation reaches a much higher frequency when the population encounters repeated drought events.

## Discussion

In the context of climate change, assessing how fast plants will adapt to longer periods of severe heat and drought stress is crucial both for conservation biology and food security (Loarie *et al*., 2009; Exposito‐Alonso *et al*., 2019; Brás *et al*., 2021). Selection from standing variation, as opposed to the emergence of new mutations (Hermisson & Pennings, 2017), is classically expected to lead to faster evolution (Barrett & Schluter, 2008). TEs however, may challenge this prediction due to the combined effect of their stress‐inducible activity and large mutagenic properties (Baduel *et al*., 2021). Here, we experimentally confirmed with a real‐time setup that a single novel insertion of *ONSEN* resulting in a loss‐of‐function of *RPI2*, can indeed rapidly lead to a selective advantage upon drought. More specifically, we document a case in which a decrease of the photosynthetic capacity under well watered conditions, by reducing plant growth and water consumption, leads to an increased survival rate under water limitation. Reducing evapotranspiration and growth are known evolutionary strategies of plants to survive drought (Kusaka *et al*., 2005; Borrell *et al*., 2014) and believed to be adaptive in ever‐drier environments (Rauschkolb *et al*., 2022). Accordingly*,* variations in the growth scaling of natural accessions of *A. thaliana* have been shown to be linked to abiotic parameters such as temperature and precipitation (Vasseur *et al*., 2018). Here, we also show that *RPI2,* the gene whose loss‐of‐function is causative for the distinct drought tolerance of hcLine31, is quantitatively associated with the aridity level in wild *A. thaliana* population.

In *A. thaliana,* several studies have established a functional link between loss‐of‐function alleles and adaptive traits (e.g. Johanson *et al*., 2000; Kroymann *et al*., 2003; Gujas *et al*., 2012) also in the context of adaptation to drought (Monroe *et al*., 2018; Xu *et al*., 2019). Together with this large body of evidence, our study substantiates the ‘less is more’ hypothesis (Olson, 1999) that is, that in contrast with intuitive expectations gene loss may fuel evolution. Indeed, in contrast with the wt, all five individuals of hcLine31 survived the severe drought stress applied in our experiment, indicating a drastic gain of fitness under these conditions. Conversely, the reduced growth of hcLine31 underlying the increased drought tolerance may be linked to a high fitness cost under more humid conditions. As our terminal drought experiment resulted in the premature death of most plants, we were not able to assess fitness‐related traits such as biomass or seed production and instead simulated under which conditions an insertion causing such a strong phenotype could be maintained in a population. We found that a severe and repeated water limitation would be needed and that such distinct, TE‐mediated adaptive effects may therefore be transient and thus difficult to capture with population genomics data. This may explain why no natural insertions of *ONSEN* were observed in *RPI2*. Conversely, positively selected loss‐of‐function mutations leading to a reduced plant size have been reported previously in *A. thaliana* (Barboza *et al*., 2013) and the absence of natural TE insertions in *RPI2* could also indicate that this transposition event did not take place in the wild or only occurred in marginal populations not yet sampled. Indeed, TE‐mediated mechanisms leading to large‐effect mutations might be especially important in less‐adapted, frequently stressed populations in which drastically altered phenotypes could be advantageous. In agreement with this hypothesis, Baduel *et al*. (2021) recently pointed towards a link between positive selection of weak alleles of the largest subunit of RNA polymerase V, a key component of RdDM, and a globally relaxed silencing of TEs in *A. thaliana* ecotypes growing under extreme conditions.

We only performed the in‐depth and labour‐intensive molecular characterisation for one of our hcLines. Therefore, whether the observed high frequency of five independent drought‐tolerant hcLines could be explained by the insertion preference of *ONSEN* towards exons and H2A.Z enriched regions that are shown to be associated with responsive genes (Coleman‐Derr & Zilberman, 2012; Quadrana *et al*., 2019; Roquis *et al*., 2021), remains speculative. Because the insertion preference of *ONSEN* has also been demonstrated in a population of epiRILs (Johannes *et al*., 2009; Quadrana *et al*., 2019), therefore by a different approach than the transient drug treatment (Thieme *et al*., 2017), we conclude that our findings using hcLines in the Col‐0 background are solid and highly relevant for the understanding the role of *ONSEN* in natural accessions. Although at a lower frequency, *ONSEN* indeed transposes in the wild (Masuda *et al*., 2016; Quadrana *et al*., 2016). Although we did not observe major phenotypic changes among the six included control lines that were only heat stressed or demethylated we can not rule out that stochastic small‐scale mutations (Liu *et al*., 2015) or reported epialleles (Roquis *et al*., 2021) caused by the drug treatment have contributed to the observed high frequency of drought‐tolerant hcLines. In this regard, other exogenous TEs such as the tobacco *Tnt1* retrotransposon (Grandbastien *et al*., 1989) that is used as a mutagen in plants including *Arabidopsis* (Lucas *et al*., 1995) and known to efficiently knock‐out genes could serve as a control in experiments systemically addressing the specific capacity of *ONSEN* to generate drought‐tolerant individuals. Whereas the comparison of the evolutionary potential of individual TE families requires a larger number of observations, our current study also provides the foundation for selection experiments with populations of hcLines whose TE‐composition has not however been shaped by natural selection. These types of experiments will allow us to extrapolate the overall gain or loss of plant fitness following a heat‐induced transposition of an endogenous TE.

In conclusion our study substantiates that stress‐induced TE mobility can rapidly lead to an increased stress tolerance of the host as formerly suggested by population genomics data (Quadrana *et al*., 2016). Because TE activity is family dependent and can be triggered by various abiotic and biotic stresses (for review Negi *et al*., 2016), these findings also support the hypothesis that TEs are more likely to rapidly modulate gene expression and traits than classical point mutations (Uzunović *et al*., 2019). In line with models of rapid adaptation through large mutation effects (Orr, 2005) and more recent works in *A. thaliana* (e.g. Fulgione *et al*., 2022) our study demonstrates that TEs can provide a powerful means by which plants can keep pace with the rapidly changing environmental conditions.

## Competing interests

None declared.

## Author contributions

Conceptualisation, MT; data acquisition, MT, BK, AB; data analysis, MT, ACR, YB; funding acquisition, MT, EB, ACR; writing – original draft, MT; writing – review and editing, MT, ACR, EB, AB, BK, YB.

## Supporting information


**Fig. S1** Leaf disc assay to validate the accuracy of the pixel counts for the machine learning‐based prediction using ilastik.
**Fig. S2** Pixel count of vital tissues in all tested lines before the occurrence of necrotic leaves and the fold change of *ONSEN* copy numbers measured using qPCR. Linear regression model.
**Fig. S3** Simulated frequency of a TE insertion in a population of *Arabidopsis thaliana* exposed to drought stress.

**Table S1** Names and sequences of oligos used for the qPCR to determine *ONSEN* copy numbers and for genotyping of *RPI2*.

**Table S2** Raw data of pixel counts and weight, SPAD values and C : N‐measurements.

**Table S3** SNPs in *RIP2* and bioclimatic variables for the subset of 596 ecotypes.
Please note: Wiley Blackwell are not responsible for the content or functionality of any Supporting Information supplied by the authors. Any queries (other than missing material) should be directed to the *New Phytologist* Central Office.

## Data Availability

Raw and segmented images and ilastik classifications were uploaded to Figshare (doi: https://doi.org/10.6084/m9.figshare.17159231). Seeds are available upon request to michael.thieme@botinst.uzh.ch.
