# Supplementary material for: Experimentally heat‐induced transposition increases drought tolerance in Arabidopsis thaliana
Source: New Phytol. 2022 Jul 9;236(1):182–94. doi: 10.1111/nph.18322 (PMC9544478; doi:10.1111/nph.18322)
Supplement: Supplementary file 1 — Fig. S1 Leaf disc assay to validate the accuracy of the pixel counts for the machine learning‐based prediction using ilastik. Fig. S2 Pixel count of vital tissues in all tested lines before the occurrence of necrotic leaves and the fold change of ONSEN copy numbers measured using qPCR. Linear regression model. Fig. S3 Simulated frequency of a TE insertion in a population of Arabidopsis thaliana exposed to drought stress. [file NPH-236-182-s004.pdf]

New Phytologist Supporting Information

Article title: Experimentally heat-induced transposition increases drought tolerance in *Arabidopsis thaliana*

Authors: Michael Thieme, Arthur Brêchet, Yann Bourgeois, Bettina Keller, Etienne Bucher, Anne C. Roulin

Article acceptance date: 10 June 2022

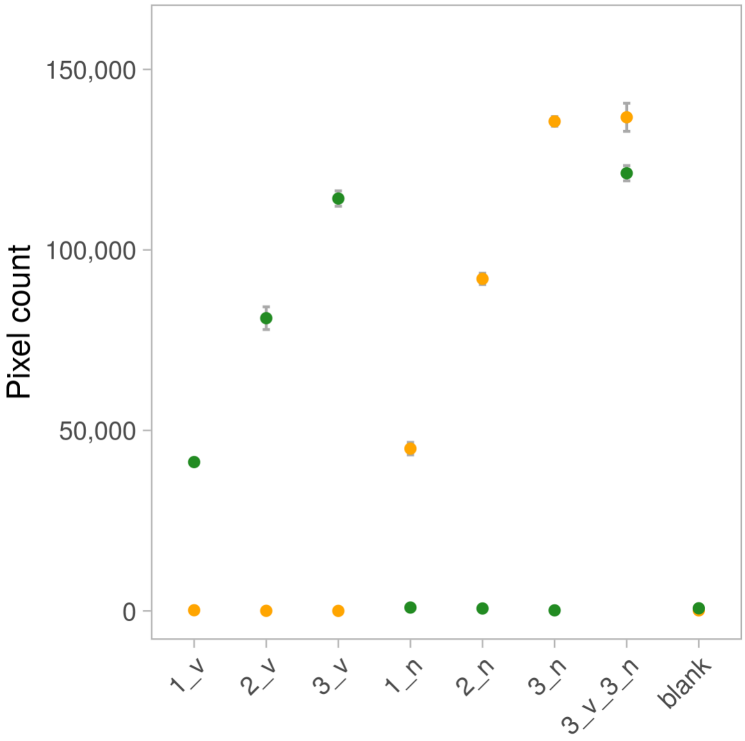

Fig. S1 Leaf disc assay to validate the accuracy of the pixel counts for the machine learning-based prediction of vital (green) and necrotic (yellow) segments using ilastik. One, two or three discs were punched from vital (v) or necrotic (n) leaf tissues and placed onto soil of an empty pot (blank). n=3 technical replicates.

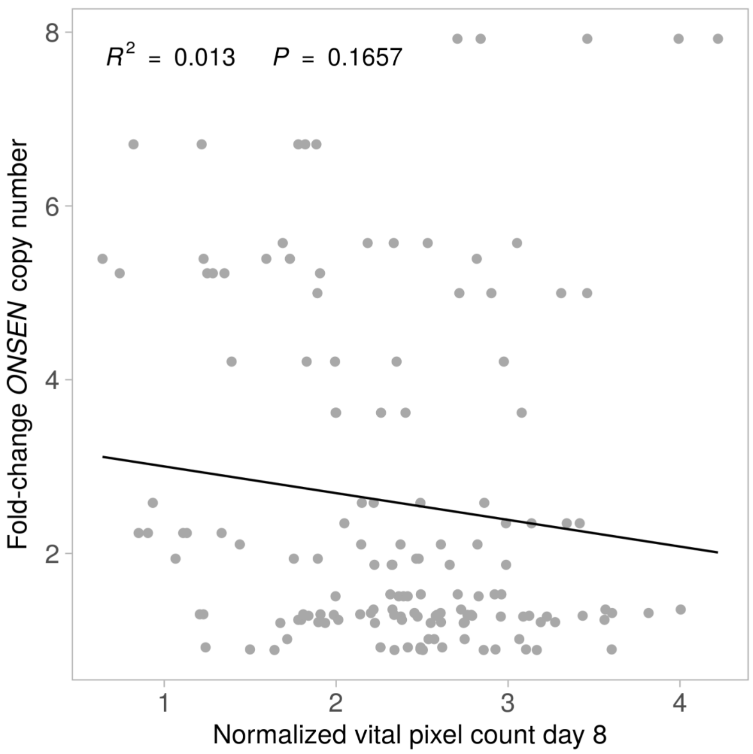

Fig. S2 Pixel count of vital tissues in all tested lines before the occurrence of necrotic leaves (day 8) (n=5 biological replicates) and the fold change of *ONSEN* copy numbers measured by qPCR relative to the wt (n=3 technical replicates). Linear regression model, adjusted R-squared: 0.013, P=0.1657.

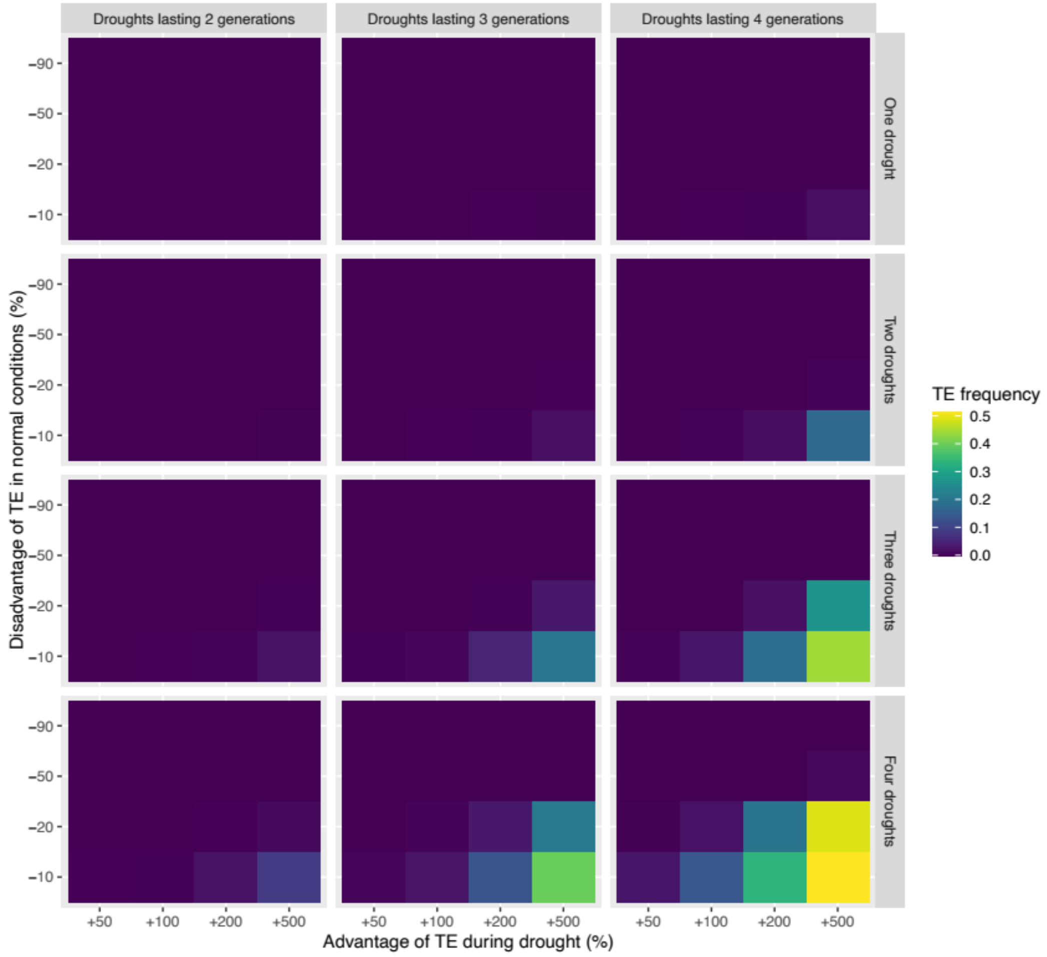

Fig. S3 Simulated frequency of a TE-insertion in a population of *A. thaliana* exposed to drought stress. Heatmap showing the average frequency of TE-insertions with differing fitness impacts under control and drought conditions in response to increasing intensity of drought stress after 50 generations. Each cell in the heatmap corresponds to 1000 simulations.
